# Multivariate data analysis of capacitance frequency scanning for online monitoring of viable cell concentrations in small-scale bioreactors

**DOI:** 10.1007/s00216-019-02096-3

**Published:** 2019-10-13

**Authors:** Sabrina Metze, Stefanie Blioch, Jens Matuszczyk, Gerhard Greller, Christian Grimm, Jochen Scholz, Marek Hoehse

**Affiliations:** 1 Sartorius Stedim Biotech GmbH, August-Spindler-Str. 11, 37079 Göttingen, Germany; 2 Leibniz University of Hannover, Welfengarten 1, 30161 Hannover, Germany

**Keywords:** Mammalian cell culture, PAT, Capacitance frequency scanning, MVDA (multifrequency permittivity, impedance spectroscopy, small-scale bioreactor, process monitoring and control)

## Abstract

Viable cell concentration (VCC) is one of the most important process attributes during mammalian cell cultivations. Current state-of-the-art measurements of VCC comprise offline methods which do not allow for continuous process data. According to the FDA’s process analytical technology initiative, process monitoring and control should be applied to gain process understanding and to ensure high product quality. In this work, the use of an inline capacitance probe to monitor online VCCs of a mammalian CHO cell culture process in small-scale bioreactors (250 mL) was investigated. Capacitance sensors using single frequency are increasingly common for biomass monitoring. However, the single-frequency signal corresponds to the cell polarization that represents the viable cell volume. Therefore single-frequency measurements are dependent on cell diameter changes. Measuring the capacitance across various frequencies (frequency scanning) can provide information about the VCC and cope with changing cell diameter. Applying multivariate data analysis on the frequency scanning data successfully enabled direct online monitoring of VCCs in this study. The multivariate model was trained with data from 5 standard cultivations. The model provided a prediction of VCCs with relative errors from 5.5 to 11%, which is a good agreement with the acceptance criterion based on the offline reference method accuracy (approximately 10% relative error) and strongly improved compared with single-frequency results (16 to 23% relative error). Furthermore, robustness trials were conducted to demonstrate the model’s predictive ability under challenging conditions. The process deviations in regard to dilution steps and feed variations were detected immediately in the online prediction of the VCC with relative errors between 6.7 and 13.2%. Thus in summary, the presented method on capacitance frequency scanning demonstrates its suitability for process monitoring and control that can save batches, time, and cost.

**Electronic supplementary material:**

The online version of this article (10.1007/s00216-019-02096-3) contains supplementary material, which is available to authorized users.

**Figure:**
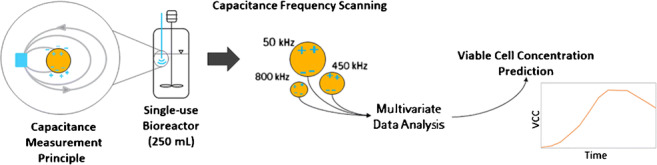
Graphical abstract

## Introduction

Mammalian cell culture processes for the production of monoclonal antibodies are challenging and complex processes that are crucial in modern medicine to generate pharmaceutical products such as anticancer drugs. Pharmaceutical companies face a strong market demand and a high pressure to provide safe products according to quality guidelines and legislations.

To improve and sustain high quality in pharmaceutical products, the International Conference on Harmonization (ICH) launched the “Quality by Design” (QbD) initiative within the framework of the ICH Q8 guideline in 2004 [1]. The QbD approach involves, inter alia, the identification of product attributes that are of significant importance to the product’s safety and establishment of robust control strategies to ensure consistent process performance [2]. The initiative focuses on the detection of critical process parameters (CPPs) that have influence on critical quality attributes (CQAs) of the product (e.g., glycosylation profile of a monoclonal antibody). Moreover, the CPPs should be monitored and controlled online to achieve a constant and high product quality.

The QbD concept of CPPs that influence CQAs in the regulatory framework can be transferred and used also in the perspective of a pharmaceutical manufacturer. Besides the CQAs, a manufacturer focuses on selected process attributes that are important for the process performance and the economy of the production. Thus, process attributes need to be monitored and key process parameters (KPPs) that influence the process attributes require to be well-controlled as well [3].

Process analytical technology (PAT) is a toolbox allowing for the implementation of QbD. When the Food and Drug Administration (FDA) launched the PAT initiative in 2004, they aimed at establishing a consistent process performance, process control, and a high product quality by monitoring and controlling KPPs or CPPs that affect process attributes or CQAs, respectively [4–6]. PAT tools are significant to fulfill regulatory needs resulting in high product quality as well as optimizing the process performance based on selected process attributes.

From a pharmaceutical manufacturer perspective, the antibody titer and the process yield are some of the most important process attributes to achieve an economic process. The viable cell concentration (VCC) reflects the amount of viable cells in a cell suspension that is responsible for the antibody production. Thus, VCC is strongly linked to product titers and is considered process attribute, too [3]. Monitoring the VCC enables process optimization and control that leads to higher titers and efficient processes. One example can be the adjustment of the feeding rate based on online VCC values leading to an optimal feed consumption in every process that reduces medium costs in the production facility. Current state-of-the-art VCC measurements comprise offline methods like trypan blue assays. Major drawbacks are the low temporal resolution of the VCC, and the temporal delay between sampling and measurement. Further, operator-dependent measurement errors can alter the results and increase risks of contaminations [7, 8]. Offline measurements for important process attributes conflict with the online control and QbD requirements biopharmaceutical processes have to meet. In recent years, many online PAT tools have been investigated in pharmaceutical processes to monitor cell concentrations of mammalian cultures (e.g., radio-frequency impedance, Raman spectroscopy, or near-infrared spectroscopy) [9–14].

Radio-frequency impedance was selected as a preferred tool for mammalian cell culture monitoring because of relatively low implementation costs, a sensitivity to cell numbers, and easy implementation into the sterile surrounding of a bioreactor [15–17]. Online radio-frequency impedance measurements as a PAT tool have been used for many years in order to measure the permittivity of mammalian cell cultivations and derive information about cell growth and biomass [15, 18–21]. Therefore, the theory and principles of radio-frequency impedance have been presented in various literature, so here only a short summary of the basic terminology and theory will be given [19, 22, 23].

In the context of radio-frequency impedance, the terms used to describe the same measurement approach differ from author to author. The same approach is described as (bio-)capacitance [15, 24, 25], dielectric spectroscopy [7, 17, 26], multifrequency permittivity [27], or impedance measurements [7].

Impedance measurements provide capacitance and conductivity as physical properties where capacitance and conductivity are generally frequency-dependent. Combined with the cell constant (which corresponds mainly to the electrode geometry), capacitance leads to dielectric properties of the analyte where the capacitance component delivers the permittivity signal measured in picofarads per centimeter of the analyte [28]. Therefore, in the following, the measurement signal of a capacitance sensor is referred to as permittivity.

**Fig. 1:**
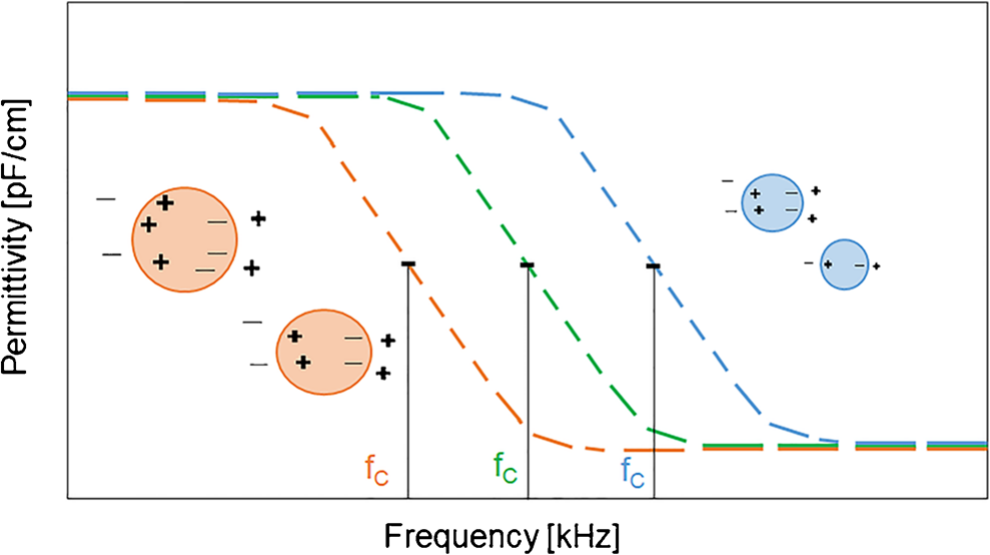
Schematic overview of a ß-dispersion curve for spherical cells. With increasing frequency, the permittivity of cells in suspension moves from a low-frequency plateau that corresponds to a maximal cell polarization down to a high-frequency plateau that corresponds to a minimal cell polarization, respectively. This typical occurrence is known as ß-dispersion. The extent of the polarization is measured by the permittivity of the electrical double layer. Depending on the polarizability of the cells, the characteristic frequency (*f*_C_) can change

When an electric field is applied to cells within an ionic solution, a charge separation occurs within the cells and the poles of the cells polarize. The polarization occurs because the electric field forces ions to move within the highly conducting cellular cytoplasm until they reach the non-conducting cellular membrane which impedes their further movement. The polarizability of the cell suspension corresponds to the permittivity of the cell suspension. This means with higher cell concentrations, more cells contribute to the polarization, leading to a higher permittivity. Furthermore, the polarizability is frequency-dependent. In the case of mammalian cells with diameters in the range of several micrometers, excitation frequencies below 100 kHz leave enough time for the cells to completely polarize, resulting in a high permittivity of the solution. An increase in the excitation frequency leads to a decrease in permittivity because the cells cannot polarize completely. This loss in cellular polarization is referred to as β-dispersion. Figure 1 illustrates the schematic overview of a β-dispersion curve for spherical cells that describes the principle of permittivity measurements. The frequency at which the rate of polarization is half complete is the characteristic frequency (*f*_C_). Depending on the polarizability and the size of the cells, *f*_C_ can change. Dead cells and impurities of the cell broth are not polarizable and therefore do not impact the capacitance measurements [19, 29]. Moreover, before inoculation of the cells, the signal of the medium is zeroed. Therefore, only changes during cultivation due to the cell culture will be detected in the permittivity signal. A cultivation broth does not consist of many polarizable species or polarizable species appear in concentrations below the limit of detection of a capacitance probe. Thus, the capacitance probe is a promising tool to monitor cell growth online.

As a result of the measurement principle, the permittivity signal measured at one frequency increases with higher VCCs in a culture as well as with a larger cell diameter that can occur during cell aging or as stress responses. The influences of VCC and cell diameter on the permittivity signal are not distinguishable with one frequency measurement. Thus, single-frequency measurements correlate with the viable cell volume (VCV) instead of the VCC [17, 30]. With radio-frequency impedance spectroscopy (here called capacitance frequency scanning), the dielectric properties of a cell suspension are measured with multiple frequencies resulting in a higher degree of cell information. Recently, it was reported that applying capacitance frequency scanning and mathematic modeling (e.g., Cole-Cole modeling of the β-dispersion curve or partial least squares regression) on the frequency scanning data revealed online information of the VCC, cell apoptosis, or nutrient limitations [24–27, 31–33].

Frequency scanning captures the permittivity at various frequencies over the course of cultivation, delivering a large amount of complex data. The interaction between different effects that influence the measurement requires advanced statistics/analysis such as multivariate data analysis (MVDA).

The handling of multivariate data and advantages of orthogonal partial least squares (OPLS) models have been well-described in literature [34–36]. Unlike PLS models, OPLS models separate the variation within the predictors into two parts: one being correlated, predictive to the model response; and one being uncorrelated, orthogonal to the model response. However, OPLS and PLS result in identical models. The main difference between OPLS and PLS is the easier interpretability when using OPLS models [35]. By definition of the OPLS algorithm, orthogonal components are orthogonal to the target information. Besides noise, they mainly cover changes due to matrix effects. Applying OPLS (or PLS) to frequency scanning data will support the predictability of VCCs as other potential polarizable species will not necessarily follow the trend over time for VCC.

This work aims to demonstrate the superiority of frequency scanning compared with single-frequency measurements in terms of VCC determination. Within this study, a new quantitative multivariate model based on OPLS regressions is developed correlating offline VCC measurements of an industrially relevant CHO fed-batch process to frequency scanning data.

As a result, the model is able to display the correct VCC throughout a cultivation reducing the need of manual sampling. Moreover, in contrast to single frequency, the multivariate model predicts the VCC in all cell growth phases including the cell death phase. Lee et al. reported a direct data-driven PLS modeling approach to achieve online VCC models for batch cultivations with low cell counts, claiming the need to verify their results in cultivations with higher cell counts such as fed-batch or perfusion processes [26]. To the best of our knowledge, this study presents for the first time a direct data-driven model based on OPLS for VCCs greater than 10 million cells/mL in a fed-batch process. Moreover, robustness of the MVDA model is investigated with fed-batches including deliberate process changes.

## Material and methods

### Cell lines and media

A DG44 CHO cell line expressing a monoclonal antibody was used in this study (Sartorius Stedim Cellca GmbH). Seed medium (SM), basal medium for production (PM), and two different feeds, i.e., feed medium A (FMA) and feed medium B (FMB), were used in all experiments (Sartorius Stedim Cellca GmbH). All media and feeds were chemically defined.

### Seed culture

The seed culture process comprised the thawing and passaging of the cells before inoculation of the bioreactor. A cryo vial of 1 mL CHO cell suspension with a concentration of 30 million cells/mL was thawed and transferred into 10 mL pre-warmed seed medium (36.8 °C) that was stored in a 15-mL falcon tube (Sarstedt). In order to remove the preservation medium, the falcon tube was centrifuged at 190*g* for 3 min (3–30K Centrifuge, Sigma). The supernatant was decanted and the pellet re-suspended in 1 mL of seed medium. The cell suspension was transferred into a single-use 0.5-L Erlenmeyer flask (Corning) containing 150 mL pre-warmed seed medium. All seed cultures were kept in an orbital shaking incubator (CERTOMAT^®^ CT plus, Sartorius Lab Instruments GmbH) at a temperature of 36.8 °C, a pCO_2_ of 7.5%, 85% humidity, and a shaking rate of 120 rpm (rpm) with an orbital diameter of 50 mm. The cells were passaged 5 times in a rhythm of 3–4 days before inoculation of the main culture.

### Main culture

The main culture was inoculated with 0.3 million cells/mL (day 0) and lasted 12 cultivation days. All experiments were carried out in a small-scale multiparallel bioreactor system (ambr^®^ 250 modular, Sartorius Stedim Biotech GmbH) with a maximum working volume of 250 mL per vessel. The process temperature set point for all experiments was set to 36.8 °C ± 0.05 °C. The pH was controlled using CO_2_ additions to maintain a pH set point of 7.1. Once a day, the pH was measured offline and re-calibrated if the result deviated by more than 0.05 units between the online and offline signals.

The set point for DO was set to 60% and the control loop adjusted the DO by modulating air and oxygen additions. The small-scale bioreactor was stirred at 855 rpm. On the inoculation day, antifoam was added depending on the actual bioreactor volume (0.001% of the total culture volume). During subsequent cultivation, antifoam was added manually depending on the foam level in the bioreactor.

FMA and FMB were added to the culture from day 3 in a ratio of 10:1 (FMA:FMB) according to Table 1.

**Table 1:** Feeding strategy of feed medium A (FMA) and feed medium B (FMB). The medium was added as volume percent of the cell broth. Day 0 was the day of inoculation

Day	3	4	5	6	7	8	9	10	11
Volume FMA (%)	3.9	3.8	3.6	3.5	3.3	3.2	3.0	2.9	2.8
Volume FMB (%)	0.39	0.38	0.36	0.35	0.33	0.32	0.30	0.29	0.28

From day 5 on, glucose was supplied as a bolus feed to the bioreactor once the glucose level dropped below 5 g/L to achieve a daily maximal glucose concentration of 5 g/L.

### Dilution experiment

The seed and main culture of the process with integrated dilution steps were conducted as described in the corresponding section. The dilution with PM amounted to 30 vol.% of the cell broth. Two dilution steps were applied at a process time of 123 h and 194 h. For the dilution, the calculated amount of cell suspension was removed by sampling of the bioreactor and the same amount of pre-warmed PM was pumped to the culture. The PM was pre-warmed in an orbital shaking incubator (CERTOMAT^®^ CT plus, Sartorius Lab Instruments GmbH) at a temperature of 36.8 °C, a pCO_2_ of 7.5%, and 85% humidity until the addition of the PM into the main bioreactor was completed. Therefore, the temperature and pH were close to process conditions. The pre-conditioning of DO was not necessary, because the main bioreactor was fully controlled and the DO set point was achieved in short process times.

### Altered feed strategy

The seed and main culture of the process with applied altered feed strategy were conducted as described in the corresponding section with exception of the feed and glucose strategy. Glucose was added on day 5 as a bolus once the glucose concentration depleted below 4 g/L to keep the set point at 4 g/L glucose. The feed control recipe of FMA and FMB was programmed according to Table 2.

**Table 2:** Feeding strategy of feed medium A (FMA) and feed medium B (FMB) for the altered feed strategy. The medium was added as volume percent of the cell broth. Day 0 was the day of inoculation

Day	3	4	5	6	7	8	9	10	11
Volume FMA (%)	5.1	5.0	4.8	4.7	4.6	4.4	4.3	4.2	4.1
Volume FMB (%)	0.71	0.68	0.66	0.64	0.62	0.61	0.58	0.57	0.56

### Offline analytics

The viable cell concentration and the viability of the cell suspension, as well as the cell diameter, were analyzed with the trypan blue assay–based Cedex HiRes Cell Counter and Analyzer system (Roche). The pH and the glucose concentration were measured offline in a blood gas analyzer (ABL800 Basic, Radiometer).

### Online capacitance measurements

Frequency scanning measurements were conducted with an impedance probe (FUTURA pico, Aber Instruments). The sensor scanned the permittivity at 25 discrete frequencies between 50 and 20,000 kHz resulting in a new measurement value for each frequency every 30 s. The probe was connected via a connection hub to a PC, and the data was processed by the FUTURA SCADA software (Aber Instruments). The data was stored as CSV format and imported into an Excel^®^ file (Microsoft Corporation) for further treatment. The single-frequency excited permittivity was measured in parallel at the frequency signal of 607 kHz via the same impedance probe. The single-frequency data was processed by the FUTURA SCADA software (Aber Instruments), and it was stored as CSV format for further data treatment in an Excel^®^ file (Microsoft Corporation).

### Data analysis and data treatment

The MVDA model was composed of frequency scanning data from cultivations and the respective VCC offline measurements. The time points when offline and online data were available were identified and summarized. The data summary was generated in Excel^®^ (Microsoft Corporation) and imported into the model building software (SIMCA^®^, Version 15, Sartorius Stedim Data Analytics). The scanning frequencies were marked as the model predictors and mean-centered. The VCC was scaled to unit variance and marked as the model response. OPLS was used to create all MVDA models. For predictions, the full dataset of the frequency scan of the selected fed-batch was imported into the OPLS model and marked as the prediction set.

Single-frequency results were analyzed in Excel^®^ (Microsoft Corporation). A linear regression was applied including the offline and corresponding online values up to a diameter change greater than 0.5 μm compared with the averaged previous diameter values was detected. Therefore, the linear regression model did not contain any values from the end of the exponential growth phase, the stationary growth phase, or the death phase. The resulted equation from the linear regression was used to predict the VCC values based on the single frequency at 607 kHz.

The predicted VCC trajectories for both approaches (single-frequency and frequency scanning) were plotted in a graphing and analysis software (Origin^®^ 2018, OriginLab Corporation). Within this software, the data were smoothed applying the Savitzky-Golay filter (second polynomial order) over a window of 30 data points.

The resulting trajectories were 1-point calibrated. As the inoculation cell concentration is usually a known parameter, the offsets of trajectories were adjusted to the corresponding inoculation cell concentration. For this purpose, the first 30 predicted VCC points were averaged after being smoothed. The difference between that averaged predicted value and the inoculation cell concentration was added or subtracted from all predicted data points of the corresponding batch.

For all predicted fed-batches, the root mean square error of prediction (RMSEP) was calculated to investigate the quality of the predicted values in comparison to the values observed with the offline reference (Eq. 1). *y*_pred_ describes the predicted VCC value based on the applied model and *y*_obs_ describes the observed VCC value based on the offline reference method. The number of observed and predicted value pairs is described by *n*.

1 $$ \mathrm{RMSEP}=\sqrt{\frac{\underset{i=1}{\overset{n}{\Sigma}}\left({y}_{\mathrm{pred}}-{y}_{\mathrm{obs}}\right)2}{n}} $$

In addition, a Batch Evolution Model (BEM) was created by importing the mean-centered permittivity values of all frequencies into the model building software (SIMCA^®^, Version 15, Sartorius Stedim Data Analytics). The BEM displays the averaged trajectory of the selected dataset, and the standard deviations serve as limits to monitor the trajectory of independent cultivations online. The trajectories displayed in a BEM are also known as golden batch trajectories. In this work, the OPLS method was used to create the BEM. The selected robustness fed-batches that were monitored with the BEM were imported as prediction sets. Thus, the setup enables an online comparison of the current fed-batch compared with the golden batch trajectory and can be used as alarm system once a deviation occurs.

### Validation of MVDA model

The OPLS model was validated applying a leave-one-batch-out (LOB) approach. Five independent models (models A–E) were created always leaving the complete dataset from one batch out that was then used as the prediction set. Table 3 summarizes the different models that were created. Each standard fed-batch served once as a prediction set and was predicted with a model comprising all other corresponding standard fed-batches. The RMSEP was calculated (models A–E in Table 3) from these predictions compared with the actual data values. The robustness trials (FB#6–8) served as prediction sets for the final model including all standard cultivations (model F in Table 3).

**Table 3:** Overview of generated MVDA VCC models

Model	Included fed-batches (FB)	RMSEC (10E6 cells/mL)	Predicted fed-batch (FB)	RMSEP (10E6 cells/mL)	Relative error (%)	*R*^2^ (%)	*Q*^2^ (%)	Principle components (predictive + orthogonal)
A	FB#2–FB#5	1.37	FB#1	1.17	6.6	95.3	95.0	1 + 2
B	FB#1, FB#3–5	1.41	FB#2	1.27	8.3	95.6	95.0	1 + 2
C	FB#1, FB#2, FB#4, FB#5	1.52	FB#3	0.99	5.5	94.3	93.8	1 + 2
D	FB#1–3, FB#5	1.27	FB#4	1.04	5.7	96.2	95.7	1 + 2
E	FB#1–4	1.00	FB#5	2.22	11.0	97.3	97.1	1 + 2
F	FB#1–5	1.36	FB#6	1.33	6.7	95.4	95.0	1 + 2
F	FB#1–5	1.36	FB#7	1.78	8.8	95.4	95.0	1 + 2
F	FB#1–5	1.36	FB#8	2.71	13.2	95.4	95.0	1 + 2

Within each OPLS model (e.g., model A), the root mean square error of calibration (RMSEC) was calculated. For this purpose, an identical calibration and validation dataset was used. Each data point of the dataset was predicted by the model resulting in the RMSEC.

## Results and discussion

### Establishment of a MVDA model using a leave-one-batch-out approach

In this work, capacitance frequency scanning was applied to an industrially relevant fed-batch process. After integration of the inline sensor into the single-use bioreactor, standard cultivations were performed to create and validate the MVDA model. The online data from the different frequencies served as predictors in the model, and the offline reference represented the response. Therefore, the presented method to correlate the permittivity information with VCC was a data-driven approach and did not need any further calculations like in previous approaches from literature (e.g., Cole-Cole modeling) [24, 25, 27].

In the following, the 5 standard fed-batch processes are analyzed (FB#1–FB#5). The standard fed-batch processes were conducted under the same culture conditions without changes to the process parameters and cultivation strategy. Typical sources of process variations as seed and medium lots differed for each cultivation. Each step followed the internal standard operating procedure (SOP) as described in the “Material and methods” section. To further understand the capabilities of the established frequency scanning MVDA model, the LOB method was applied. Table 3 summarizes the prediction results and the RMSEP for each cultivation being discussed in the following chapters.

**Fig. 2:**
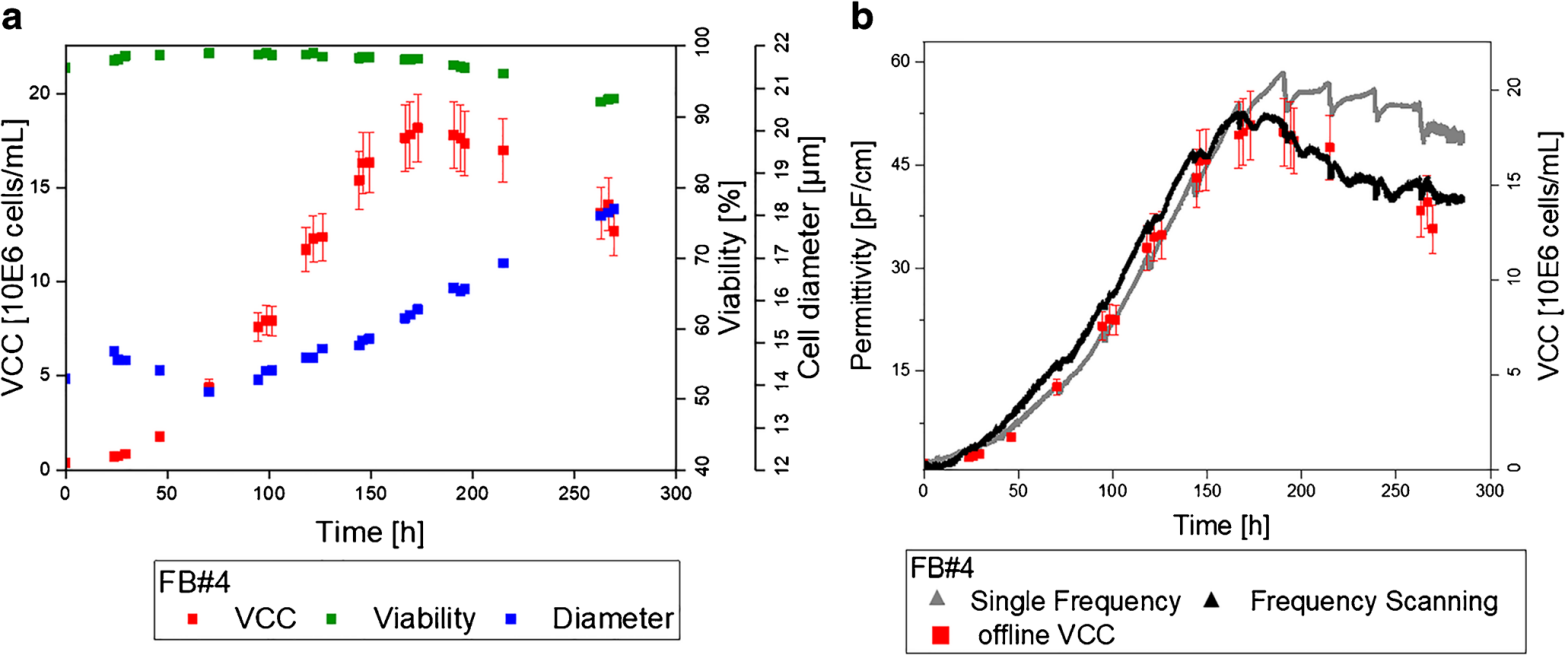
Results of the standard fed-batch cultivation FB#4. **a** Viable cell concentration (VCC), viability and cell diameter trajectories for the investigated fed-batch process over a cultivation time of 12 days (288 h). **b** Corresponding online permittivity signal (single frequency), the prediction based on the multivariate model (frequency scanning) and the offline reference VCC values for FB#4. The indicated error bars for the offline reference VCC values describe the prediction error acceptance criterion of 10%

Figure 2 a represents the VCC trajectory and other process parameters of one selected fed-batch (FB#4). The cell growth represented in VCC started with a short lag phase (0–48 h), followed by an exponential growth phase with a peak cell concentration of 18 million cells/mL (48 –168 h) and finally reached the stationary and death phases at the end of the process. At the end of the exponential growth phase (for FB#4 after 150 h), a strong increase of the cell diameter was detected in all standard fed-batches as exemplarily demonstrated in Fig. 2 a. With inoculation of the main bioreactor, the viability kept constantly high until day 8 of cultivation. After that day, the viability decreased to 92% for FB#4. The end viabilities for all standard fed-batches were in the range of 87% and 93% (see Electronic Supplementary Material (ESM) Table S1). The peak cell concentrations, end cell concentrations on day 12, the end viability, and the average cell diameter increase from day 0 to day 12 are summarized in Table S1 (see ESM) for all standard fed-batches.

Figure 2 b represents the predicted VCC trajectories of FB#4 based on the permittivity of one frequency and on the MVDA VCC model based on frequency scanning using the LOB method. The VCC prediction based on the MVDA model represents the VCC offline values over the complete cultivation time within the accepted error range of 10% of the offline reference method. The authors assume the measurement error of the widely used and accepted offline method to be at least 10%. This conservative estimation is based on experimental experience and is in agreement with previous literature reports [14]. However, for further discussion, 10% error is taken as acceptance criterion for the MVDA VCC model. Table 3 indicates the RMSEP and the relative errors for the prediction of all standard fed-batches within the LOB method. In this study, the RMSEP varied between roughly 1 and 2 million cells/mL corresponding to relative errors between 5.5 and 11% for the fed-batches FB#1 to FB#5 (Table 3). The coefficient of determination for all presented models was above 94%. Therefore, the prediction error of the frequency scanning model matches the uncertainty of the offline reference method. Thus, frequency scanning is a suitable technique for online monitoring of VCC and facilitates the implementation of advanced control strategies.

The online single-frequency permittivity trajectory and the trajectory based on the VCC prediction model reveal the great potential of the measurement approach itself (Fig. 2b): Each feed addition can be monitored online resulting in dips, starting with the first feed after 72 h of cultivation time. Moreover, the impact of the feed in the permittivity signal increased over time resulting in stronger dips in the stationary and apoptotic phases. The feed dips in the permittivity signal of single-frequency measurements were showing a decrease in the VCC higher than the pure dilution effect of the cells. It can be concluded that the permittivity signal might include information about metabolic cell changes. This effect was described by Ansorge et al. who were able to correlate feed-related changes in the permittivity to the cell metabolism [18]. However, this result needs to be confirmed in future experiments. The MVDA trajectory did not show such strong feed dips at the end of the cultivation. The OPLS algorithm excludes orthogonal information (noise) that does not correlate to the VCC. Therefore, the cross-sensitivity to other cell metabolic effects is reduced.

The permittivity trajectory of the single-frequency measurement correlated with the offline VCC within the exponential growth phase up to a significant cell diameter change (Fig. 2b). In the apoptotic cell culture phase at the end of the cultivation, the permittivity and the offline values were no longer correlated. The strong increase in the cell diameter resulted in a higher VCV and therefore a higher single-frequency permittivity signal compared with the VCC reference. This result is in good agreement with previous publications and confirms the expectations based on the literature [17]. The next section provides a more detailed comparison of the single-frequency measurements versus the MVDA analysis of the frequency scan.

### Comparison of the MVDA model and single-frequency measurements

In analogy to the MVDA analysis for the frequency scanning, the LOB method was applied to the single-frequency measurements. Therefore, five separate linear regressions consisting of 4 fed-batches each were created. The individual left out fed-batch served as prediction set. The single-frequency measurement at 607 kHz and the offline VCC reference up to a cell diameter change greater than 0.5 μm were used to create a linear regression model. The criterion of the cell diameter change was selected based on the previously discussed literature indicating an impact of the cell diameter on single-frequency measurements [17]. The selected criterion was in agreement with observations concerning the cell diameter behavior in all standard fed-batches that can be seen exemplarily in Fig. 2 a for FB#4. The total diameter change of the cells over the complete cultivation time ranged from 1.9 up to 4.6 μm (see ESM Table S1). The equation from the linear regression model was used to predict the VCC of each fed-batch. Table S2 (see ESM) gives an overview of the different linear regressions presented in this work.

**Fig. 3:**
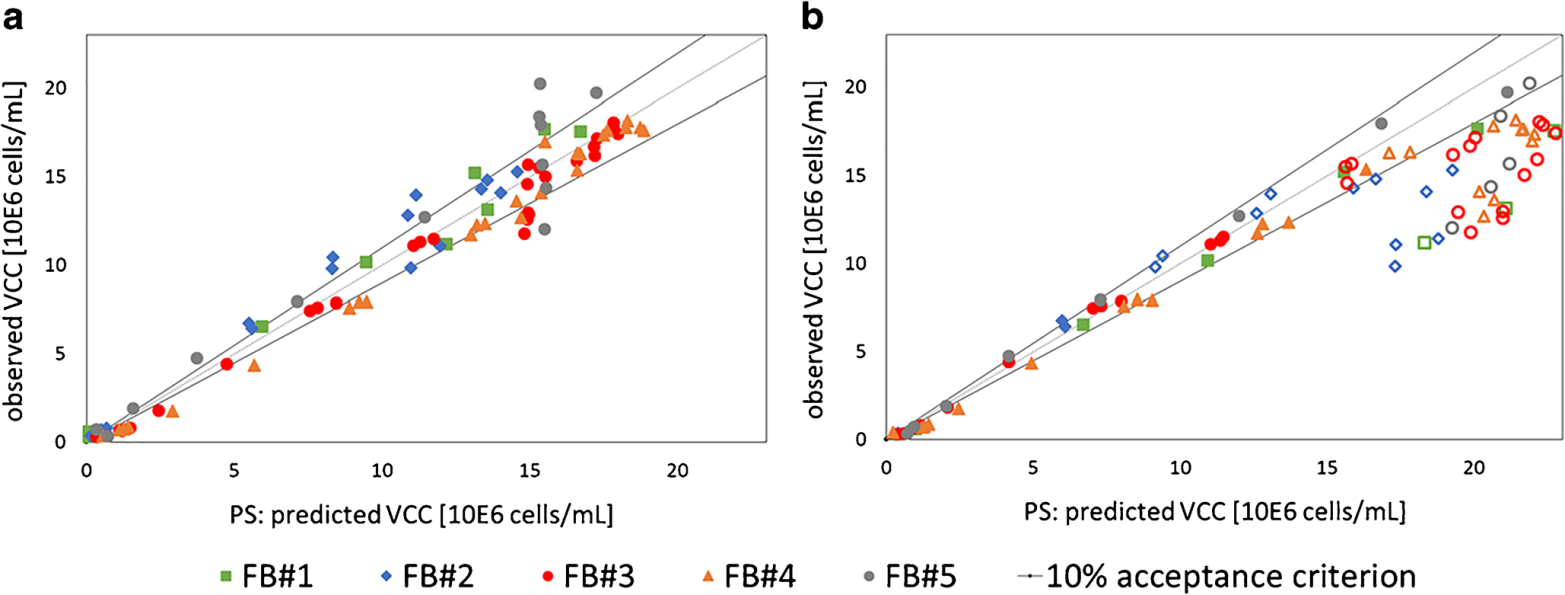
Observed versus predicted plot. **a** Comparison of the observed viable cell concentration (VCC) and the predicted VCC from the prediction set (PS) based on the leave-one-batch-out (LOB) method for the multivariate data analysis. **b** Comparison of the observed VCC and the predicted VCC from the PS based on the LOB method for the single-frequency correlations. The empty symbols reflect the measurement values that were excluded from the correlations based on linear regression

In Fig. 3, the predicted VCCs for each fed-batch using the LOB method are plotted against the VCC reference data points for the frequency scanning VCC model (Fig. 3a) and for the single-frequency correlations (Fig. 3b). The dotted straight line through the origin with the same distance to the *x*-axis and *y*-axis at every point corresponds to a perfect correlation between the observed and predicted values. The gray lines in the figure indicate the 10% acceptance criterion for the VCC prediction. A prediction within this error range is comparable to the current state-of-the-art measurement method and indicates a successful implementation of the online sensor approach for VCC predictions. In the MVDA model, there was a strong consistency between predicted and observed VCC values with few exceptions for all tested standard cultivations (Fig. 3a). As already mentioned previously, the RMSEP for the multivariate analysis resulted to be between 1 and 2 million cells/mL with relative errors between 5.5 and 11% for the fed-batches FB#1 to FB#5 (Table 3).

Figure 3 b displays the results of the LOB method applied to the single-frequency measurements. However, the single frequency did not provide a correlation within the 10% acceptance criterion over the complete cultivation time. In the exponential growth phase below VCCs of 15 million cells/mL, a good correlation was given. With higher VCCs, the observed and predicted values were not comparable to each other and out of the 10% acceptance range. Empty symbols indicate the VCC values that were not included into the linear regression because of the diameter change greater than 0.5 μm.

The RMSEP for the single-frequency analysis (between 3 and 4 million cells/mL) was much higher compared with the RMSEP values achieved with the MVDA model (Table 3 and ESM Table S2). The relative error for the prediction based on single frequency ranged from 15.8 to 22.7%. The accuracy of the single-frequency measurement was not comparable to the offline reference method.

When dealing with such numbers, one should keep in mind that single-frequency measurements do not correlate to VCC if cell diameter changes occur [17, 30]. Therefore, the error of this method is strongly dependent on the data point selection and distribution. Many data points within the death phase of the cells (with higher cell diameter) will increase the error values for VCC predictions automatically as they were not considered in the linear regression (empty symbols). Using linear regression models to predict the values in the death phase necessarily results in high deviations. To better compare the single-frequency VCC prediction and the MVDA VCC model, the RMSEP of the values after a significant cell diameter change of 0.5 μm that served as criterion for the linear regression was calculated. Table S3 (see ESM) summarizes the RMSEP and relative errors for the VCC values after the cell diameter change. The superiority of the frequency scanning MVDA model compared with the single-frequency prediction is clearly demonstrated for the stationary and death phases of the cells. The RMSEP for the MVDA VCC model ranged from 1 to 3 million cells/mL (rel. errors between 5.4 and 15.2%) whereas the RMSEP for the single-frequency prediction was calculated to be between 4 and 6 million cells/mL (rel. errors between 25 and 34%). The relative error of the VCC prediction in the cultivation period with an increased diameter was only for the MVDA model inside the acceptable range with one exception of FB#5 with 15.2% that was only close to the acceptance criterion. The exception is probably caused by VCC variations within the standard process and a small amount of cultivations in the calibration set. The results can likely be improved by a larger set of calibration runs in future.

To conclude, the results of the LOB method demonstrate that the MVDA model provides significant benefits compared with single-frequency measurements in predicting VCCs over the complete culture time for the presented fed-batch process. The frequency scanning itself is a powerful tool for monitoring the process attribute VCC with a high accuracy and with reduced cross-sensitivity to cell diameter changes. For future applications, use of frequency scanning in combination with MVDA for VCC online monitoring is therefore recommended.

### MVDA model properties including all standard cultivations

The previous section demonstrates that the MVDA model based on standard cultivations predicts the VCC of an independent fed-batch with a high accuracy. For future applications, the MVDA model will be trained and extended with every successful cultivation. In accordance with future applications, the most robust model containing all 5 cultivation training sets is described and used for further analysis and robustness trials.

**Fig. 4:**
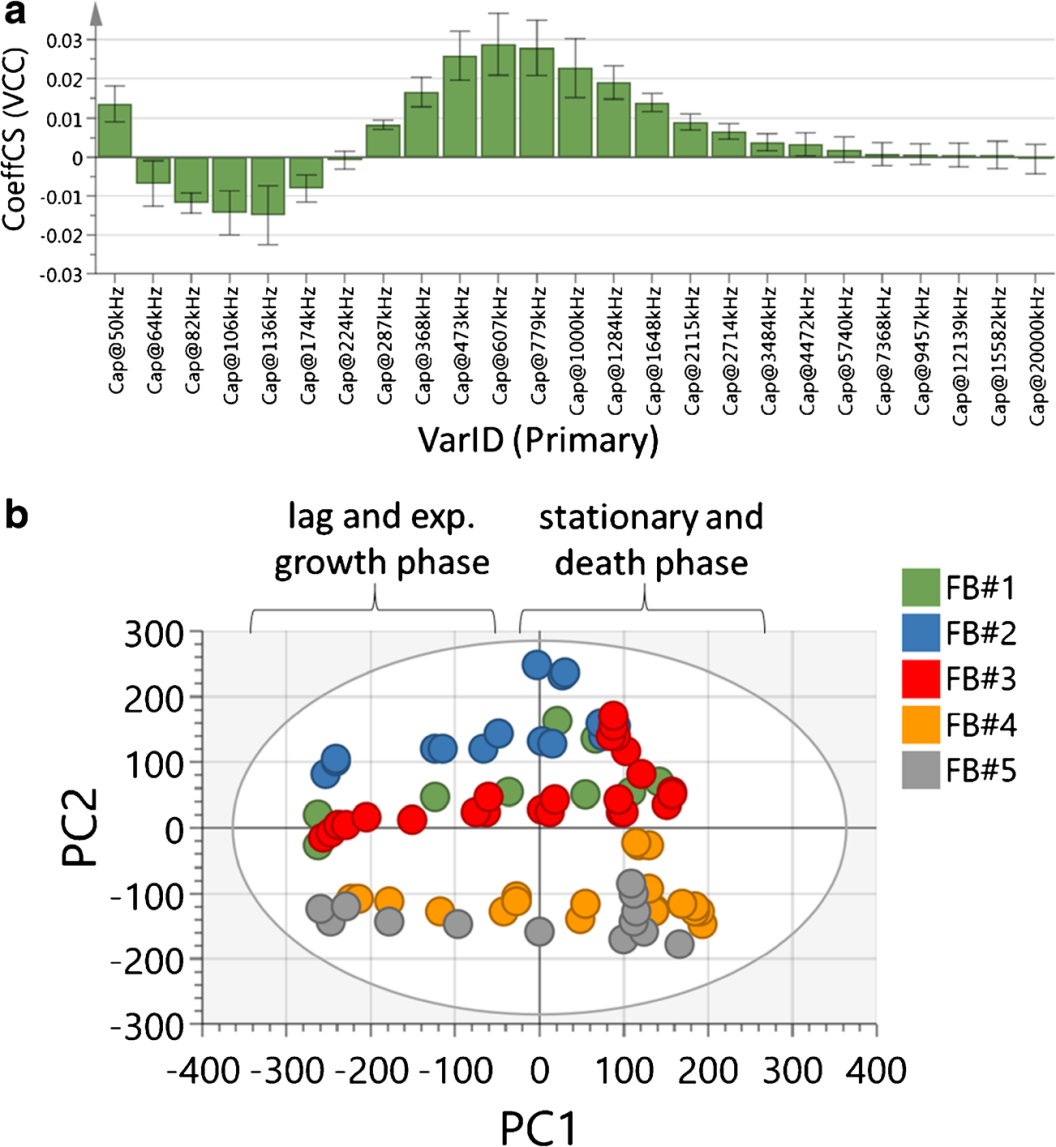
Overview of the multivariate model containing all standard fed-batches FB#1 to FB#5. **a** Contribution of each of the 25 measured frequencies to the multivariate model. **b** Score plot indicating the distribution of the fed-batch cultivations (FB#1–FB#5) dependent on the predictive principle component (*x*-axis) and the first orthogonal principle component (*y*-axis). The ellipse around the plotted data points indicates the 95% confidence bound based on Hotelling’s T2 statistics

Figure 4 a shows the regression plot and therefore the influence of each frequency on the MVDA VCC model. The frequencies can be divided into 3 different regions that are indicated by different hills or valleys. Within each region, the resolution of the permittivity measurement is different. The information within one valley or hill might represent the same effect in different resolutions. Therefore, the information might correlate to the same effect within one region. In the investigated frequency range, three affected sub-ranges are revealed. The three different sub-ranges support the amount of selected principle and orthogonal components of the MVDA model (Table 3). For all MVDA VCC models, 1 predictive and 2 orthogonal components were used. In addition to the regression plot (Fig. 4a), the *R*^2^/*Q*^2^-summary of fit plot supports the selection of components (see ESM Fig. S2). It is common to stop adding principle component in case an additional component does not result in an improvement of more than 10%. Moreover, the comparison of *R*^2^/*Q*^2^ and RMSEC/RMSEP (Table 3) should be roughly similar. Based on the presented results, 1 predictive and 2 orthogonal components were used for all MVDA VCC models (Table 3).

In comparison to single-frequency measurements at 607 kHz, the result of three different sub-ranges indicates that frequency scanning delivers additional information in lower frequencies. This additional information is included in the MVDA model and improves the predictability of VCC values. The analysis of the score contribution plots in a point-to-point comparison of two data points within one fed-batch underlined the increase of information in lower frequencies compared with the single frequency at 607 kHz. Figure S1 (see ESM) demonstrates the score contribution plot for each frequency in a point-to-point comparison for the cultivation days 5 and 12 in FB#3. The VCC values on the selected days were in a comparable range (11.51 million cells/mL for day 5 and 11.79 million cells/mL for day 12), but a strong change in the cell diameter was detected (14.7 μm for day 5 and 18.4 μm for day 12). A strong effect at low frequencies, especially at 50 kHz, was identified. Therefore, the information from low frequencies might include information about the cell diameter changes in the late exponential growth phase, the stationary phase, and the death phase.

The use of multiple frequencies and a MVDA VCC model resulted in a higher accuracy of VCC predictions especially in the death phase of each cultivation (Fig. 3). Furthermore, in future applications, it might be possible to follow the approach to reduce the amount of detection frequencies from 25 frequencies down to one frequency per identified valley or hill. A reduction of frequencies enables an improved process control by a higher measurement frequency and results in smaller databases for the online calculations.

Figure 4 b shows the score plot including all standard cultivations regarding the first two principle components (PC1 and PC2). PC1 is the predictive component located on the *x*-axis, and PC2 is the first orthogonal component on the *y*-axis. Each fed-batch is colored individually. The scores of each cultivation can be separated based on the cultivation time of each score. The scores in the lag phase and the exponential growth phase did not distribute in PC2 within one fed-batch. However, the scores of each fed-batch in the declining phase distributed in PC2. In agreement with the statements above, this result indicates that the MVDA model improves the VCC predictability especially during the death phase because of additional information described by further PCs based on different frequencies. Moreover, the score plot displays the distribution of the standard cultivations that were carried out. Each standard cultivation started from a separate seed train and was carried out at a different time leading to different medium charges and other variances. All these variances represented the accepted variation according to SOPs in the presented fed-batch process. The process parameters were not changed between runs. However, a variation within PC2 from process to process was detected. FB#2 and FB#5 were divided and distinguishable with an offset in the PC2. This difference was also reflected by comparing the RMSEC for the model containing FB#1 –FB#4 (1 million cells/mL) and the resulting RMSEP (2 million cells/mL) of FB#5 (Table 3). Thus, for FB#5, the RMSEP was more than doubled compared with the RMSEC of the corresponding multivariate model. Therefore, FB#5 seems to be an important fed-batch that needs to be included into the MVDA model even though FB#5 was performed according to the same protocol as all other fed-batches. This result is in agreement with the higher relative error for FB#5 that was previously detected when predicting the VCCs only after the cell diameter change of 0.5 μm (see ESM Table S3). To conclude, there are variations from process to process that should be accounted for in the model building process. A sufficient number of standard cultivations are necessary to create a robust and optimized MVDA model that is able to describe variations between standard fed-batches according to SOPs. This is in good agreement with other publications which stated that a robust model should contain at least 5 batches [16, 26]. In this work, a single-use, small-scale bioreactor was used for all standard cultivations. The benefit of using small-scale, single-use systems is fast turnover times with low experimental costs. Therefore, this approach provides a rapid and an economic method to achieve a sufficient number of cultivations for a robust MVDA model that can be used for future online monitoring and control of the process. The automated small-scale bioreactor enables the possibility to develop a robust model in one single run using several bioreactors at the same time and setting up a design of experiment (DoE). Thus, the presented approach can be applied in early process development where DoEs and multiparallel small-scale bioreactors are commonly used. However, the scalability of the established MVDA model to other bioreactors should be investigated in the future. The application of the MVDA VCC model in large-scale bioreactors is critical to move from early process development towards production. Therefore, it might be possible that the MVDA VCC model needs to be further developed during scale-up by including larger bioreactor scales into the model. This approach is commonly used for advanced models and leads to strong and robust predictions of the target value.

### Robustness test —dilution series

The robustness trials were applied to ensure that the prediction of the MVDA VCC model is not based on any correlation to standard process behavior or process time. The first robustness trial consisted of two dilution steps of the cell broth during one cultivation (FB#6). The cell broth was diluted by 30 vol.% with pre-warmed PM. The advantage of using a dilution with PM as first robustness trial was to implement simple changes to the permittivity signal without significantly influencing the process parameters and cell biology. Therefore, the change in the permittivity was expected to be related predominantly to the dilution steps itself and therefore the VCC changes.

**Fig. 5:**
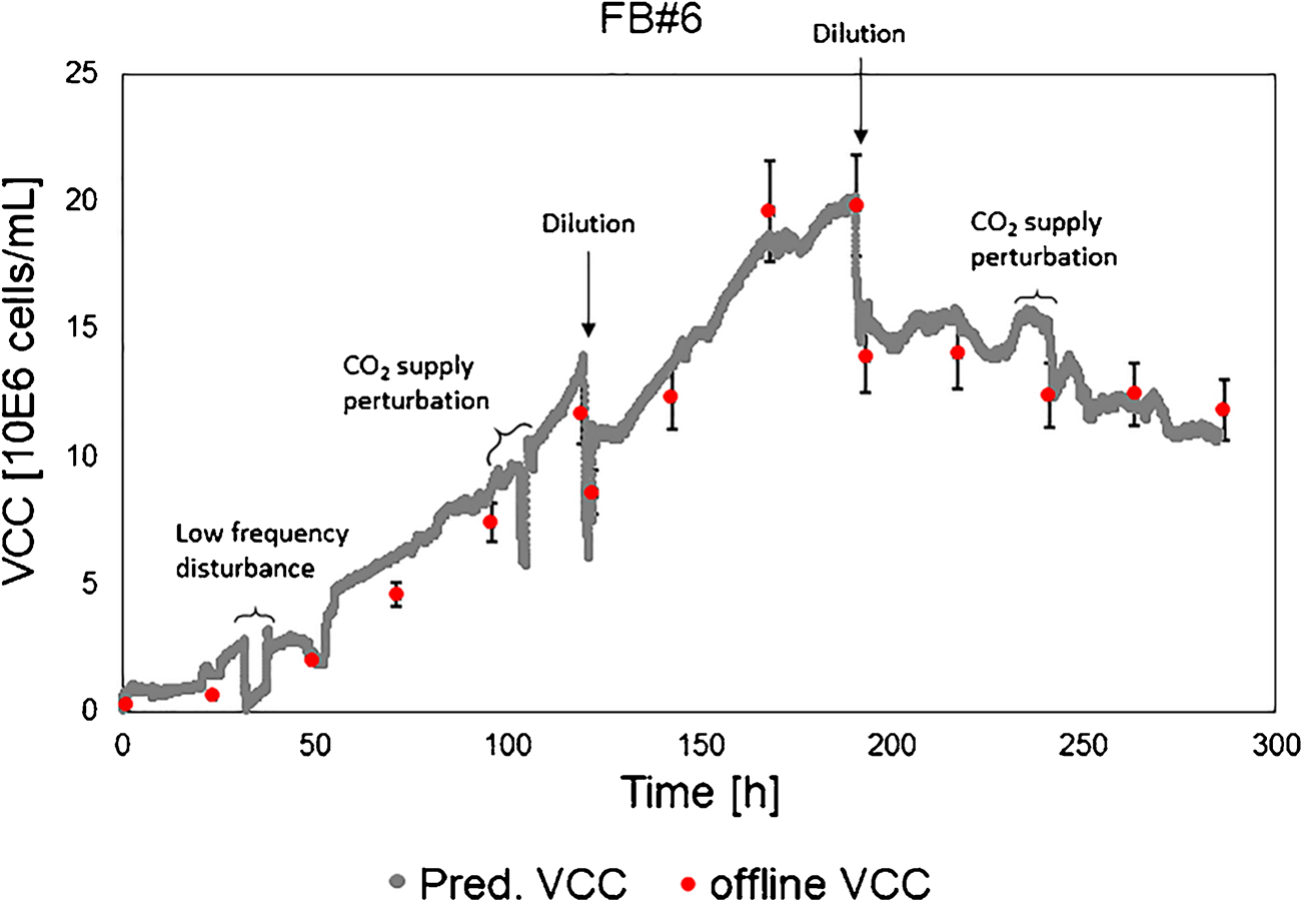
Prediction results based on the multivariate model including all standard cultivations for the dilution robustness trial (FB#6). Dilutions were applied after 123 h and 195 h of cultivation time. The online prediction of the viable cell concentration (VCC) was compared with the offline reference method. The indicated error bars for the offline reference VCC values describe the prediction error acceptance criterion of 10%

The dilutions were applied after 123 h and 194 h of cultivation time (Fig. 5). As described previously, cell diameter changes can have an impact on the permittivity signal. Therefore, the timing of each dilution was selected to cover process changes in the exponential growth phase (constant cell diameter) and in the stationary phase (incl. cell diameter changes), respectively.

Besides a low-frequency disturbance (probably caused by grounding issues of the used setup with temporarily electromagnetic interference) and CO_2_ supply perturbations, the dilution steps were detectable in the online signal (Fig. 5). The CO_2_ supply perturbations had no apparent effect on the complete cultivation and can be seen as unintended robustness tests. The influence of the CO_2_ supply perturbations on the cells was visible in the permittivity signal, leading to an increased real-time process understanding on the cellular level. Each offline reference sample that was measured after the dilution steps was matching to the predicted online VCC value. Moreover, the RMSEP of the MVDA VCC model for the complete cultivation was calculated to be 1 million cells/mL (Table 3). The prediction resulted in a relative error of 6.7% for FB#6. The predicted VCCs for the diluted fed-batch process were within the 10% acceptance criterion and comparable to the offline reference. Moreover, process disturbances (e.g., CO_2_ supply perturbations) were detected in the online signal immediately. Therefore, the online signal can be used for early fault detection. Such an alarm system will enable fast corrective actions to minimize process deviations that might lead to process failures and lost batches.

This result indicates that the MVDA model based on the 5 standard cultivations is a robust tool for VCC online monitoring. The process changes were detected immediately even though no induced process variation was previously included in the MVDA VCC model.

### Robustness test—varying feeding strategy

The dilution in the previous robustness trial was selected as being a significant process deviation with direct impact on the VCC and immediate detection in the offline reference method. To further investigate the MVDA model regarding its behavior towards less obvious process deviations, different feeding strategies were applied to the standard cultivation. The feed was slightly decreased compared with the standard feeding strategy (Tables 1 and 2). Even small process disturbances (e.g., different nutrient levels in the cell culture) in the exponential growth phase might influence the investigated process attribute (VCC) in a later stage of the cultivation or the quality attributes of the product itself [37 –40]. Thus, the feed robustness trial aims at investigating the model robustness with changes having an indirect impact on the cell growth. For this purpose, a multivariate BEM based on the frequency scanning data and deviations in the univariate offline parameter were compared.

**Fig. 6:**
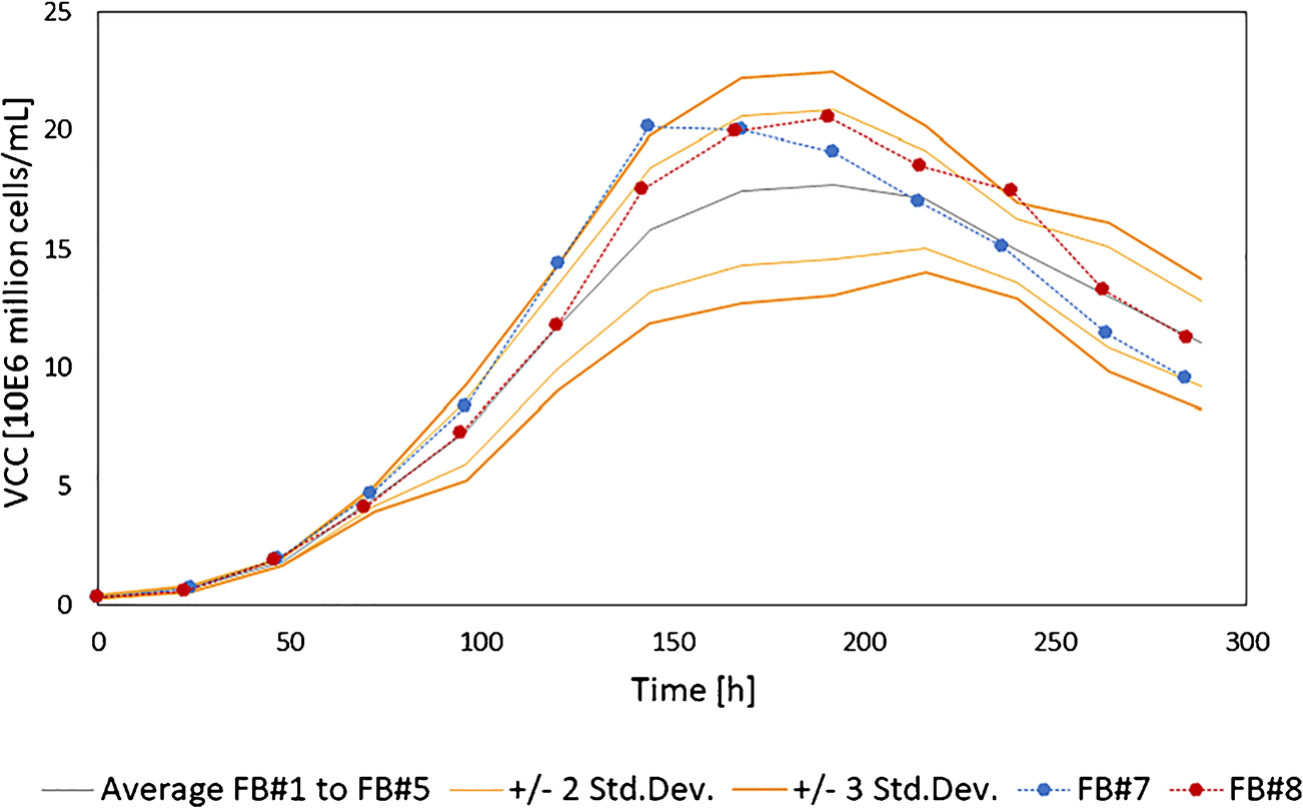
Comparison of viable cell concentration (VCC) offline reference values of the feed variated processes (FB#7 and FB#8) and the standard cultivations (FB#1–FB#5). The VCC values for the standard cultivations were averaged and the 2 and 3 standard deviations were plotted

A process control chart of the offline VCC for FB#1 to FB#5 was generated applying two respectively three standard deviations as control limits (Fig. 6). This control chart was used to compare the offline VCC of the feed variation trials FB#7 and FB#8. The offline VCC values were mainly inside the range of two standard deviations for the complete culture time. FB#7 showed two outliers outside the two standard deviations after 120 h and 144 h of cultivation time. However, remembering the minimum of 10% acceptance criterion, these outliers are not considered critical for the processes. Moreover, in the literature, deviations up to 3 standard deviations are a common acceptance criterion [41]. Applying three standard deviations to the presented offline reference results in no critical outliers for any of the two batches. For FB#8, the offline VCC was within the two standard deviations for the complete cultivation time except of the measurement value at 240 h that was still within the three-standard-deviation range. For both cultivations, no consecutive outliers were detected. Consecutiveness of outliers can serve as trigger for alarm functions and reduces the likelihood of false alarms due to single-event faulty measurements. In summary, offline VCC measurements did not detect induced process variations based on the varying feeding profiles.

**Fig. 7:**
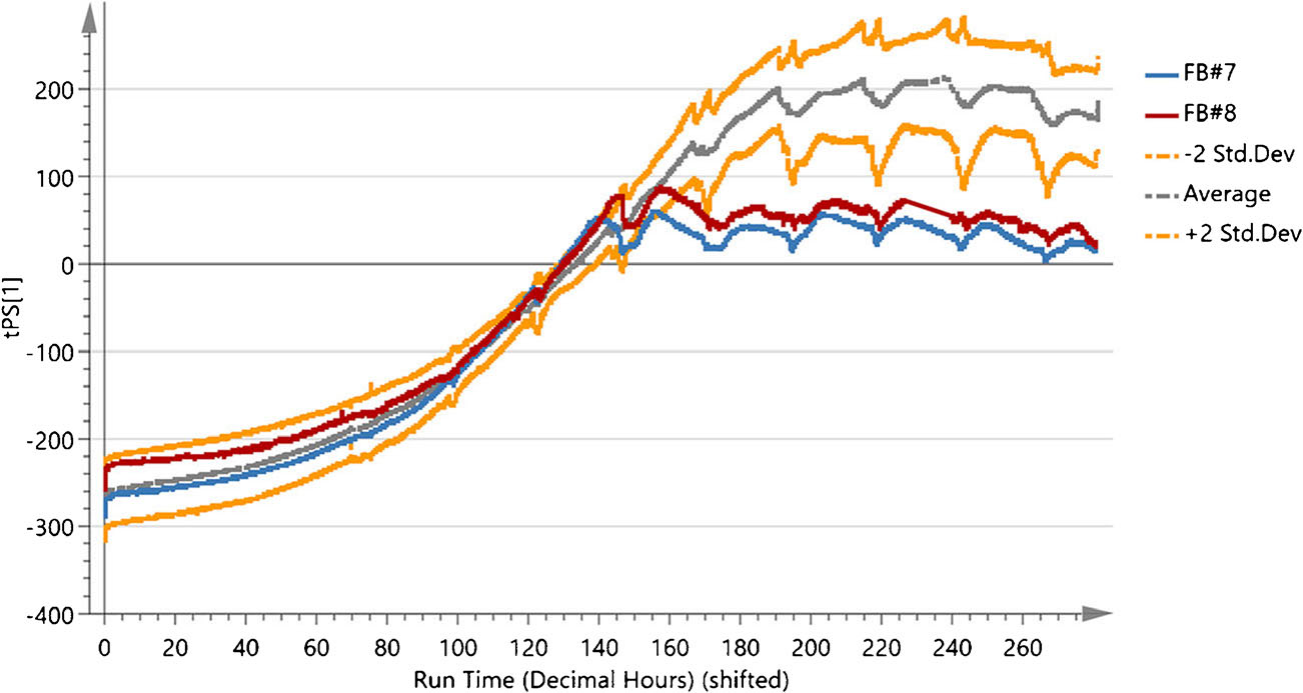
Batch Evolution Model (BEM) created from all standard cultivation (FB#1–FB#5). The BEM displays the golden batch trajectory based on the zeroed permittivity data for all frequencies. The zeroed permittivity frequency scans of the fed-batches with a varied feed strategy (FB#7 and FB#8) were included as prediction sets and monitored online in the BEM over the complete cultivation time

The online monitoring capabilities were tested by applying the BEM to the fed-batches with the altered feeding profile (Fig. 7). The multivariate BEM displays an average trajectory for the frequency scanning data dependent on the process time with two standard deviations. Compared with the offline reference in Fig. 6, a strong deviation in both altered feed fed-batches (FB#7 and FB#8) was detected. After 140 h of cultivation, both fed-batches were outside the alarm limits of two standard deviations. Compared with the univariate control chart based on offline VCC reference, the BEM allowed for a significantly earlier detection of the induced process deviation. Therefore, applying the online monitoring enables a fast identification of process deviations in regard to the investigated process attribute (VCC).

Applying MVDA and using the offline reference VCC values to build the model are an indirect measurement method. The absolute error of the reference method rises with increasing VCC. Therefore, the VCC prediction in the death phase of the cell culture results inevitably in higher prediction errors compared with the beginning of a process. Considering a common reference VCC measurement device (in industry and academia), the error of the MVDA VCC model is comparable to the reference uncertainty over the complete culture time and can be equally used for decision-making processes in the death phase of a cell culture.

Table 3 summarizes the predictions of FB#7 and FB#8 based on the MVDA VCC model including all standard cultivations (FB#1 –FB#5). The relative errors of the prediction were calculated to be 8.8% for FB#7 and 13.2% for FB#8. Both fed-batches were predicted with low errors, and FB#7 was below to the 10% acceptance criterion for VCC predictions even though the MVDA model used for the predictions was simply based on standard cultivations only. The relative error of FB#8 was slightly higher but still comparable to the offline reference system error. The prediction accuracy and model robustness can likely further be improved by including the process robustness trials into the MVDA model in future.

In summary, combining MVDA and frequency scanning can lead to be a powerful tool for future control strategies with implemented alarms saving batches, costs, time, and resources. Furthermore, the use of the single-use, small-scale bioreactor enables investigations of purposely induced process deviations by reducing medium and maintenance costs for each cultivation (including the dilution trial and the changed feed strategies). This result leads to the conclusion that the MVDA VCC model provides high accuracy even with changing process conditions.

## Conclusion

Within this work, the superiority of frequency scanning over single-frequency measurements was demonstrated, particularly during the death phase of the investigated fed-batch process. The relative errors for the data points ranged between 5.4 and 15.2% for the MVDA VCC model after the cell diameter changed (more than 0.5 μm compared with the averaged previous cultivation days). In contrast to this, the single-frequency VCC prediction resulted in relative errors between 25 and 34%. Including the complete cultivation time, the MVDA model predicted the VCC with relative errors between 5.5 and 11% in standard cultivations. The robustness of the MVDA model was successfully proven resulting in relative errors between 6.7 and 13.2%. A BEM model provided immediate information about process deviations that were difficult to detect in the corresponding offline reference on its own.

The use of a small-scale, single-use bioreactor provided a fast and economic development of a robust MVDA model with a sufficient amount of standard cultivations. Moreover, the use of the small-scale bioreactor enabled the proof of model robustness with deliberately induced process deviations. In the small scale, medium and maintenance costs for each cultivation were reasonably low.

To conclude, the combination of a capacitance probe as an inline monitor tool with MVDA enabled predictions of the VCC as a major process attribute for cell cultivation processes. Thus, the method presented here is recommended for future monitoring and control strategies such as feed control or the endpoint determination of a cultivation process based on online VCC predictions. The various monitoring and control possibilities of the presented method lead to economic, safe, and robust mammalian cell culture processes according to FDA’s PAT and QbD approaches.

## Electronic supplementary material

ESM 1 (PDF 274 kb)
