# Supplementary material for: Multivariate data analysis of capacitance frequency scanning for online monitoring of viable cell concentrations in small-scale bioreactors
Source: Anal Bioanal Chem. 2019 Oct 13;412(9):2089–102. doi: 10.1007/s00216-019-02096-3 (PMC8285309; doi:10.1007/s00216-019-02096-3)
Supplement: Supplementary file 1 — (PDF 274 kb) [file 216_2019_2096_MOESM1_ESM.pdf]

Analytical and Bioanalytical Chemistry

Electronic Supplementary Material

Multivariate data analysis of capacitance frequency scanning for online monitoring of viable cell concentrations in small-scale bioreactors

Sabrina Metze, Stefanie Blioch, Jens Matuszczyk, Gerhard Greller, Christian Grimm, Jochen Scholz, Marek Hoehse

Table S1 Summary and comparison of standard Fed-Batch cultivations used for MVDA model

Fed-Batch (FB)	Peak VCC [10E6 cells/mL]	End VCC [10E6 cells/mL]	End Viab. [%]	Diameter change Day0 –Day12 [µm]	Comment
FB#1	17.71	11.19	87.4	3.61	Standard Fed-Batch
FB#2	15.30	9.83	89.4	4.61	Standard Fed-Batch
FB#3	18.10	12.92	93.0	3.77	Standard Fed-Batch
FB#4	18.17	12.67	92.6	3.37	Standard Fed-Batch
FB#5	20.26	12.04	87.2	4.02	Standard Fed-Batch
FB#6	19.88	11.83	94.3	4.10	Dilution during standard Fed-Batch
FB#7	20.16	9.52	67.4	1.96	Altered feed strategy
FB#8	20.56	11.21	94.6	1.90	Altered feed strategy

Table S2 Overview of prediction results based on the single-frequency measurements

Linear regression	Included Fed-Batches (FB)	Predicted Fed-Batch (FB)	RMSEP [10E6 cells/mL]	Relative Error [%]
A	FB#2-FB#5	FB#1	4.02	22.72
B	FB#1, FB#3-5	FB#2	3.47	22.67
C	FB#1, FB#2, FB#4, FB#5	FB#3	3.86	21.38
D	FB#1-3, FB#5	FB#4	3.38	18.59
E	FB#1-4	FB#5	3.20	15.82

Table S3 RMSEP for the MVDA VCC model and single-frequency VCC prediction for values only after the significant diameter change greater than 0.5 µm

		MVDA VCC model		Single-frequency VCC model	
Included Fed-Batches (FB)	Predicted Fed-Batch (FB)	RMSEP [10E6 cells/mL]	Relative Error [%]	RMSEP [10E6 cells/mL]	Relative Error [%]
FB#2-FB#5	FB#1	1.31	7.38	6.02	33.98
FB#1, FB#3-5	FB#2	1.46	9.58	4.17	27.27
FB#1, FB#2, FB#4, FB#5	FB#3	1.30	7.29	5.28	29.18
FB#1-3, FB#5	FB#4	0.99	5.43	4.81	26.49
FB#1-4	FB#5	3.07	15.17	5.07	25.04

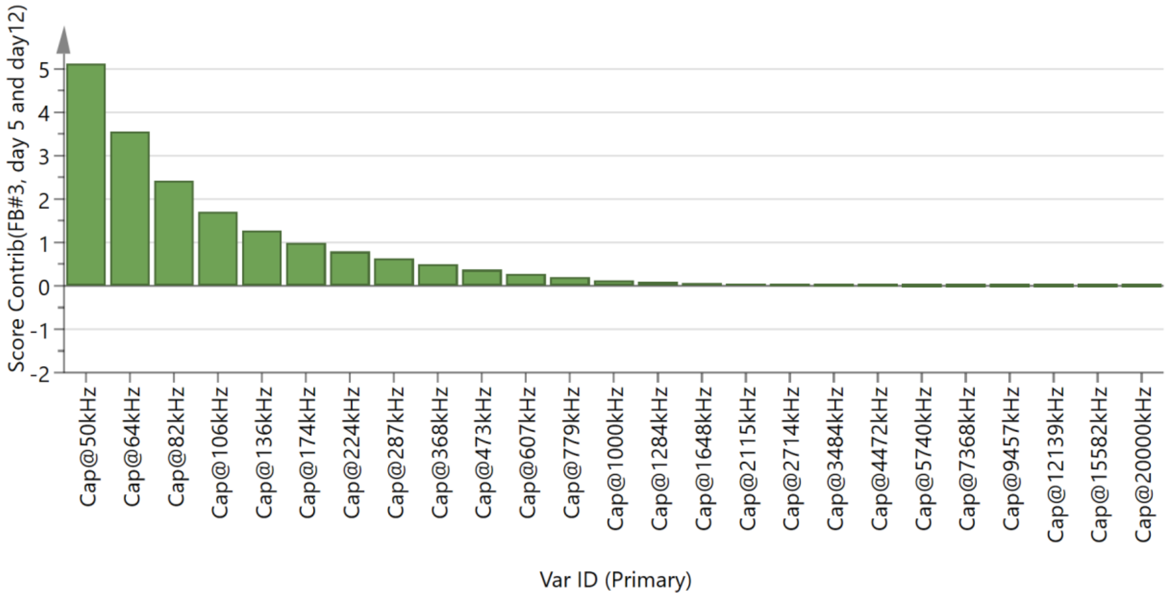

Fig. S1 Score contribution plot of the point to point comparison of day 5 and day 12 for fed-batch #3. The Viable Cell Concentration (VCC) values for both days were in a comparable range (11.51 million cells/mL for day 5 and 11.79 million cells/mL for day 12) but the cell diameter changed during the cultivation from 14.7 µm on day 5 to 18.4 µm on day 12

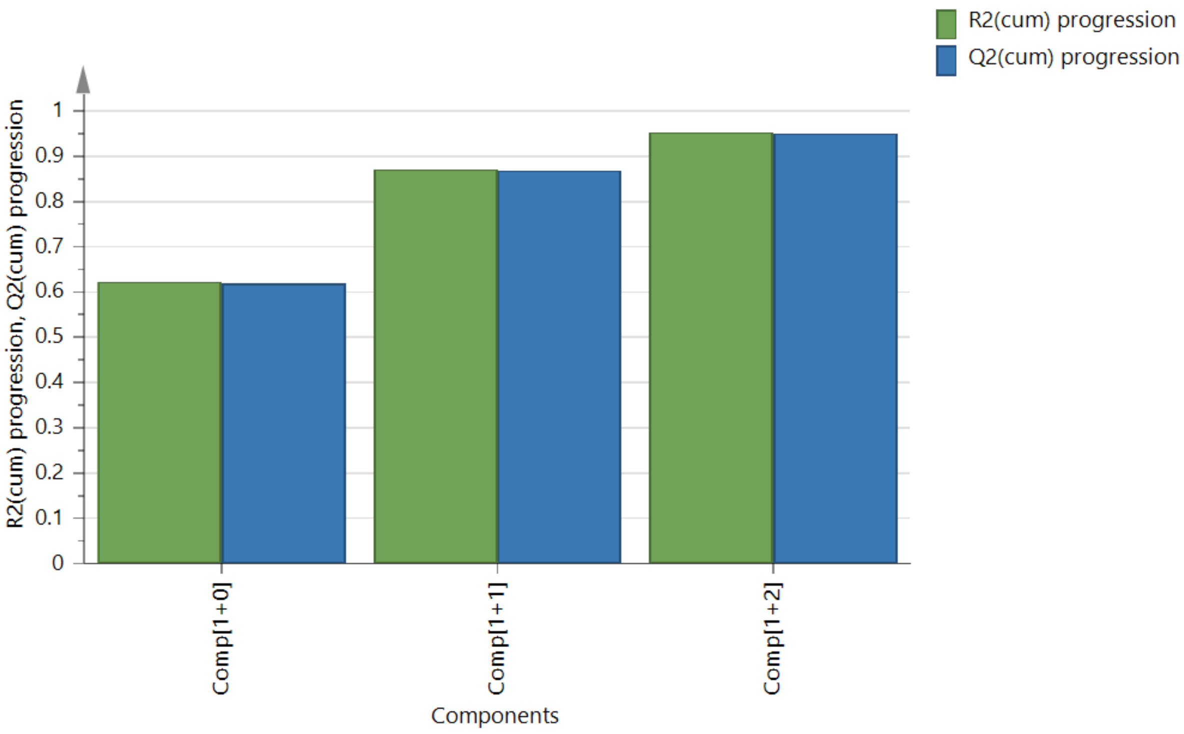

Fig. S2 Summary of Fit Plot for the MVDA VCC model F containing all standard cultivations. The plot represents the model predictability based on the selection of principle components
